# ICGRM: integrative construction of genomic relationship matrix combining multiple genomic regions for big dataset

**DOI:** 10.1186/s12859-019-3319-y

**Published:** 2019-12-26

**Authors:** Dan Jiang, Cong Xin, Jinhua Ye, Yingbo Yuan, Ming Fang

**Affiliations:** 1 Key Laboratory of Healthy Mariculture for the East China Sea, Ministry of Agriculture and Rural Affairs, Fisheries College, Jimei University, Xiamen, 361021 Fujian Province China; 2 Institute of Dermatology and Department of Dermatology, the First Affiliated Hospital of Anhui Medical University, Hefei, 230032 China; 3 College of Science, Heilongjiang Bayi Agricultural University, Daqing, 163319 China

**Keywords:** Genomic relationship matrix, Genomic selection, Gblup

## Abstract

**Background:**

Genomic prediction is an advanced method for estimating genetic values, which has been widely accepted for genetic evaluation in animal and disease-risk prediction in human. It estimates genetic values with genome-wide distributed SNPs instead of pedigree. The key step of it is to construct genomic relationship matrix (GRM) via genome-wide SNPs; however, usually the calculation of GRM needs huge computer memory especially when the SNP number and sample size are big, so that sometimes it will become computationally prohibitive even for super computer clusters. We herein developed an integrative algorithm to compute GRM. To avoid calculating GRM for the whole genome, ICGRM freely divides the genome-wide SNPs into several segments and computes the summary statistics related to GRM for each segment that requires quite few computer RAM; then it integrates these summary statistics to produce GRM for whole genome.

**Results:**

It showed that the computer memory of ICGRM was reduced by 15 times (from 218Gb to 14Gb) after the genome SNPs were split into 5 to 200 parts in terms of the number of SNPs in our simulation dataset, making it computationally feasible for almost all kinds of computer servers. ICGRM is implemented in C/C++ and freely available via https://github.com/mingfang618/CLGRM.

**Conclusions:**

ICGRM is computationally efficient software to build GRM and can be used for big dataset.

## Background

Majority of economic traits in animal and human diseases have polygenic nature, the precise estimate of genetic value is important for animal breeding and disease-risk evaluation in human. Henderson [1] proposed a best linear unbiased estimate (BLUP) to estimate genetic value, which takes advantage of the inheritance similarities among individuals based on pedigree information. With the development of sequencing technique, using whole genome-wide SNPs to calculate the similarities among individuals has been well developed [2], in which the pairwise kinship among individuals is usually described with a matrix called genomic relationship matrix (GRM). The method using GRM instead of pedigree to estimate breeding value is called genomic best linear unbiased estimate (GBLUP), which has been widely applied to estimate breeding value in the animal breeding program for dairy cattle and pig [3–5] instead of BLUP method.

Recently, whole-genome re-sequencing technique has been applied to genomic selection, by which millions of SNPs can be genotyped. Although it is able to increase the accuracy of breeding-value estimate, the computational RAM is very demanding. Furthermore, for some species, such as dairy cattle, usually animals from multiple breeding farms and countries are combined for breeding value prediction, which increases the RAM requirement cubically. Therefore, it is very meaningful to develop new software to solve this problem.

Sophisticated statistical method plays an important role on discovery of important biology law, especially in the genomic era [6, 7], it also helps to accelerate the computational speed or decrease the computer memory [8]. Current method for GRM construction is largely based on VanRaden’s algorithm [9–13], although it is mathematically simple, the computer RAM cost is very big especially when the number of individual and SNPs are huge. However, we found the equation of VanRaden’s algorithm is additive for both numerator and denominator. It motives us to factorize VanRaden’s equation into two parts and calculates numerator and denominator respectively. Taking advantage of such additive character, we are able to use split and combine strategy to reduce the computational burden.

We herein develop a package, called ICGRM, which splits the genome SNPs into several parts and calculate the summary statistics for each part that only needs very few computer RAM; then combines the summary statistics for each part to produce GRM. ICGRM avoids calculating GRM for the whole genome at the same time; thus it makes the construction of the GRM more efficient for big dataset. The other feature of ICGRM is that it can assign a weight for each SNP effect in the construction of the GRM, by which it further increases the prediction accuracy for breeding value estimate.

### Implementation

We use Intel Math Kernel Library (Intel MKL) with BLAS routine to perform matrix operation. BLAS implementations are optimized for speed on a machine in parallel, which greatly reduces the computational time. We wrap the algorithm to computational package ICGRM, which is implemented with a command line script, including two parts, GRM calculation and integration routines. The command lines to achieve the task are below.

**./clgrm_grm --in_file <inputFile> --weight_file <inputWeightFile> --out_file <outputFile> -–threads_num <numberOfThreads>**

**./clgrm_combine <inputFileList>**

The first command line is to construct GRM matrix for a specific genome segment, by which ICGRM calculates GRM for each segment separately, where users can optionally define the weight of SNP effect with “**--weight_file**”; and the second command line is to combine each GRM from each segment/loci for generation of the final GRM.

## Results

**Fig. 1:**
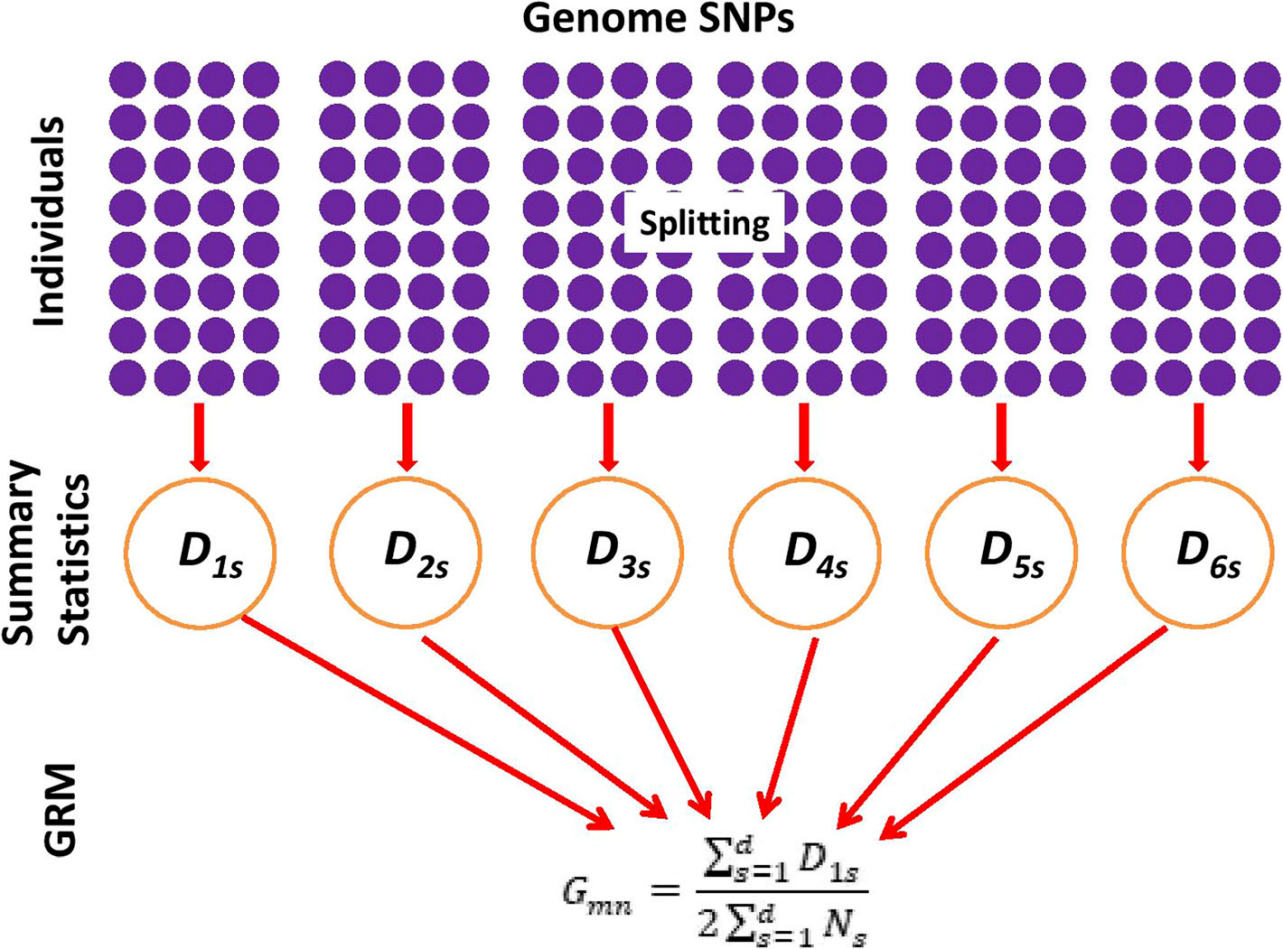
The flowchart of the ICGRM

The idea of the proposed method is that it firstly splits the genome SNPs into *d* segments, for each segment, it calculates the summary statistics related to each GRM; finally, it combines these summary statistics to produce the GRM (see Fig. 1).

### Integrating summary statistics to GRM

Suppose there are *k* SNPs in genome, in VanRaden’s equation (VanRaden 2008), the GRM is expressed as:

1 $$ G=\frac{ZZ^{\prime }}{2{\sum}_{j=1}^k{p}_j\left(1-{p}_j\right)} $$

Specifically, for two individual *m* and *n* with *K* SNPs investigated, the relationship for individual *m* and *n* can be expressed as

2 $$ {G}_{mn}=\frac{\sum_{j=1}^K\left({x}_{mj}-2{p}_j\right)\left({x}_{nj}-2{p}_j\right)}{2{\sum}_{j=1}^K{p}_j\left(1-{p}_j\right)}=\frac{\sum_{j=1}^K{z}_{mj}{z}_{nj}^{\prime }}{2{\sum}_{j=1}^K{p}_j\left(1-{p}_{\mathrm{j}}\right)} $$

Both numerator and denominator is the sum over statistics of *K* SNPs, respectively, which motivates us to split *k* SNPs into *d* segments, each containing *k*_1_, *k*_2_, ⋯, and *k*_*d*_ SNPs, respectively, and *K=* ${\sum}_{s=1}^d{k}_s$. Then we calculate the two statistics for each segment. Specifically, for segment *s* with *k*_*s*_ SNPs, we calculate ${D}_s=\sum \limits_{j={k}_{s-1}}^{k_s}{z}_{mj}{z}_{nj}^{\prime }$ and ${N}_s={\sum}_{j={k}_{s-1}}^{k_s}{p}_j\left(1-{p}_j\right)$, respectively, for *s* = 1, 2, ⋯, *d* and *k*_0_ = 1. After calculating these summary statistics, we save them on computer disk, and then use them to calculate GRM for whole genome using the eq. (3).

3 $$ {G}_{mn}=\frac{\sum_{s=1}^d{D}_s}{2{\sum}_{s=1}^d{N}_s} $$

### Weighted GRM with SNPs

As suggested by Fragomeni et al. (2017), the accuracy of the GBLUP can be further increased by taking the weight of SNP effect into account and the weighted GRM can be written as

4 $$ G=\frac{ZDZ^{\prime }}{2{\sum}_{j=1}^k{p}_j\left(1-{p}_j\right)} $$

where, *D* is a diagonal matrix with the diagonal entry *d*_*j*_ being the weight of each SNP effect. For two individual *m* and *n*,

5 $$ {\mathrm{G}}_{mn}=\frac{\sum_{j=1}^K\left({x}_{mj}-2{p}_j\right){d}_j\left({x}_{nj}-2{p}_j\right)}{2{\sum}_{j=1}^K{p}_j\left(1-{p}_j\right)}=\frac{\sum_{j=1}^K{z}_{mj}{d}_j{z}_{nj}^{\prime }}{2{\sum}_{j=1}^K{p}_j\left(1-{p}_j\right)}, $$

which shows that the numerator and denominator are also additive for each SNP or segment, suggesting that our split-combine strategy is also applicable for this case. We also add this function into our package ICGRM for ease of use.

### Simulations

**Fig. 2:**
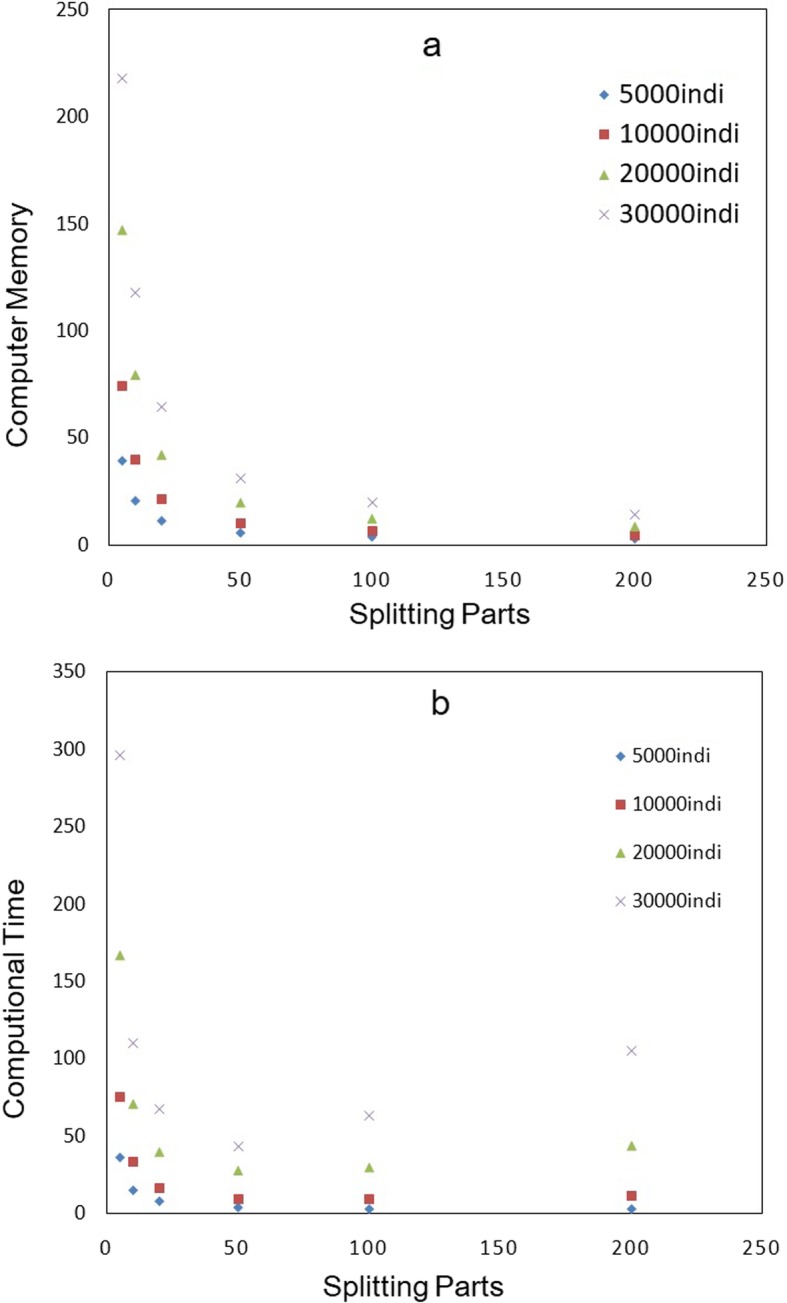
The computational memory (**a**) and time cost (**b**) at different number of individuals and splitting parts

To illustrate the performance of the proposed method, we simulate a genome with the number of individuals from 5000 to 30,000; and the number of SNPs ranged from 0.1 to 10 million. As shown in Table 1, when the number of SNPs is greater than 10 million and the number of individuals is greater than 10,000, the computational memory cost is greater than 250G, which has already been difficult for many computer servers to run the job and we also do not complete the job due to computer memory limitation. We therefore employ ICGRM to achieve this. For comparison, we keep using 10 CPU threads for calculation although we can use more. We conduct an experiment using datasets with 10 million SNPs and 5000, 10,000, 20,000, 30,000 individuals, respectively. To decrease the memory use, for each setup, we split dataset into 5, 10, 20, 50, 100 and 200 parts, respectively, in terms of SNP number; we calculate summary statistics for each part, separately, and then integrate them together to produce GRM. The memory and computational time cost are shown in Fig. 2a. It can be seen that when the dataset is split into more parts, the memory cost decreased dramatically (~ 15 times reduction from 5 to 200 parts); specifically, when the individual number is set at 30,000, the memory cost reduces from 218Gb to 14Gb, making it computationally feasible for almost all kinds of computer server. We also summarize the total computational time in Fig. 2b. It shows that when data are split into 5 to 50 parts, the total computational time cost is reduced about 7 times (using server with Intel Xeon CPU and total RAM 384Gb); but when the number of splitting parts is greater than 50, the computational time is starting to increase, and most of the computational time is consumed during data writing process.

**Table 1:** The computer memory cost at different level of individuals and SNPs

	0.1 Million SNPs	0.5 Million SNPs	1 Million SNPs	10 Million SNPs
5000	3. 9G	11.4G	20.7G	181.2Gb
10,000	6.4G	21.2G	39.9G	–
20,000	12.3G	42.1G	79.4G	–
30,000	19.8G	64.5G	118.3 G	–

## Discussion

We have developed a computational efficient algorithm and software ICGRM, which is useful for big dataset. When the number of SNPs is greater than 10 million and number of individual is greater than 10,000, the current method is not able to run due to the computer memory limit, but ICGRM solves this problem by splitting the dataset and merging the summary statistics, which reduces the computer memory dramatically. The software will be useful in future for prediction of genomic breeding value for big dataset.

## Conclusions

ICGRM is computational efficient software to build GRM and can be used for big dataset.

## Availability and requirements

**Project name:** CLGRM.

**Project home page:** https://github.com/mingfang618/CLGRM

**Operating system(s):** Linux.

**Programming language:** C++.

**License:** No.

**Any restrictions to use by non-academics:** Communication with author.

## Data Availability

The simulation datasets used in the study are available from the corresponding author.
